# The Lived Experience of Australian Women Living with Breast Cancer: A Meta-Synthesis

**DOI:** 10.31557/APJCP.2019.20.11.3233

**Published:** 2019

**Authors:** Lalithambigai Rajagopal, Pranee Pranee, Kate A. McBride

**Affiliations:** 1 *Translational Health Research Institute, *; 2 *School of Science and Health, *; 3 *School of Medicine, Western Sydney University, Penrith, New South Wales, Australia. *

**Keywords:** breast neoplasm, life experiences, social support, qualitative research, decision, making, Australia

## Abstract

**Background:**

Breast cancer is the second most common cancer among Australian women. In 2019, an estimated 19,000 women in Australia were diagnosed with breast cancer, with around 3,058 women dying from the disease in the same year. Although many qualitative studies published in Australia exist which examine breast cancer from various perspectives, only limited literature is available which addresses Australian women’s lived experience of breast cancer from diagnosis, treatment and beyond.

**Method:**

Meta-synthesis of qualitative studies. Participants who took part in either semi-structured interviews or surveys with open-ended questions were included. A thematic synthesis analysis approach was used.

**Results:**

Five themes and 13 sub themes emerged from the data analysis which illustrated the lived experience of Australian women diagnosed with breast cancer. Emotional burden and women’s response towards their breast cancer diagnosis were key themes. Experience of decision- making , social distress, symptoms beyond changes in their body, fertility considerations and their role as mothers were some of the challenges during their treatment. Women coped and adjusted with these challenges through the support of their family, and healthcare providers. Women developed greater empowerment by making their life choices after treatment. Life choices such as getting into a new relationship was challenging for single women.

**Conclusion:**

Although most women were emotionally supported following their diagnosis, there are still areas where women could be better supported such as when having to break the news of their breast cancer diagnosis to their children, provision of ongoing emotional support for caregivers of women with breast cancer, providing constant emotional and informational support at the point of diagnosis and during their treatment, tailoring treatments according to different stages of pregnancy, and discussion of fertility treatments in timely manner by healthcare professionals.

## Introduction

Breast cancer is one of the most common causes of mortality among women globally and is most commonly diagnosed cancer among Australian women (Australian Institute of Health and Welfare [AIHW], 2019). The risk of developing breast cancer before the age of 85 years old is one in eight in Australia. In 2019, it is estimated that ~19,000 women in Australia will be diagnosed with breast cancer, with 3,058 women dying from the disease (AIHW, 2019). The overall breast cancer survival rate for women in therefore high with a five-year survival rate of 90.8% and ten-year survival rate of 83%, though breast cancer in pre-menopausal women has poorer outcomes than in older post-menopausal women (Breast Cancer Netwrok Australia [BCNA], 2019). 

Over the last 20 years, multiple qualitative studies have been published in Australia examining breast cancer from various perspectives. We found multiple studies on breast cancer experiences from both psychosocial and medical perspectives. We also found studies on the meaning of breast cancer, as well as coping challenges during treatment and interpretations of mortality in the context of breast cancer. A few studies focussing on breast cancer experiences for mothers, rural and young women or within a specific cultural context were also found. Internationally, several studies have examined the lived experiences of women diagnosed with breast cancer. For example, Perreault and Bourbonnais (2005) explored the experience of six Canadian women living with breast cancer, Joulaee et al., (2012) examined the lived experience of breast cancer of Iranian women across all ages and Cebeci et al., (2012) examined Turkish women’s lived experience while receiving their chemotherapy treatment. However, only limited literature was available which addressed Australian women’s lived experience of breast cancer and their feelings explicitly from diagnosis, treatment and beyond. 

Given the uniqueness of the Australian healthcare system and the country’s unique demographic characteristics in terms of multiculturalism, understanding the lived experience of Australian women with breast cancer in this meta-synthesis may allow health care providers in the Australian healthcare system to understand these experiences to a greater depth. This in turn may help facilitate greater sensitivity when treating their patients. This is particularly important given that the complexity and intensity of breast cancer treatment has increased in recent years, thereby imposing greater challenges on the lives of women undergoing treatment for breast cancer (Stout et al., 2012). Health care providers need to understand the meaning of this lived experience of breast cancer from the perspective of Australian women in order to design appropriate interventions to reduce and/or prevent distress in these women in the unique Australian healthcare setting. 

The aim of this meta-synthesis is therefore to draw on the findings of qualitative research studies that have investigated breast cancer to provide a better understanding of Australian women’s experiences of the disease and associated treatments, thereby incorporating women’s views and perspectives to inform recommendations for healthcare policy and practice as well as future research. 

## Materials and Methods

*Inclusion and exclusion criteria*

For articles to be included in this meta-synthesis, the study needed to meet the following criteria: (a) be related to lived experiences of female individuals with breast cancer (b) utilised a qualitative methodology (c) the patients’ perspectives were explored (d) the sample were from and living in Australia (e) was published in English. Grey literature was excluded.

*Search strategy*

**Figure 1:**
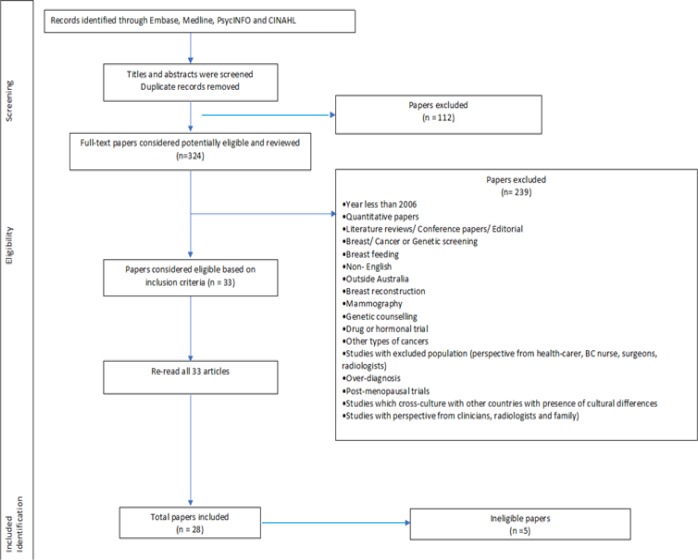
Search Strategy

A broad electronic search of the literature was conducted using Medline, Embase, CINAHL and PsycINFO between May 2017 and September 2017. Key words were included in the search in combination with MeSH (medical subject headings). The initial search found 436 articles (172 from Medline, 141 from Embase, 101 from PsycINFO and 22 from CINAHL), with 324 articles remaining for consideration once duplicates were removed (Figure 1). 

*Screening of papers*

Two authors (LR and KM) independently screened all 324 studies’ titles and abstracts according to the relevance of their methodology and themes. If the abstract did not provide sufficient information, the full text was read to determine its eligibility. Differences were resolved during team meetings. A challenge was whether or not to include mixed methods studies. After discussion with all authors, we decided not to include any mixed methods studies, except one study, that involved non – open ended questionnaires as they do not contribute any themes, and thus excluded them from analysis. We included one mixed study by Perz et al., (2014) as their online survey involved two open-ended questionnaires which contributed to five themes. 

*Assessment of Papers - Quality Appraisal*

Evaluating the quality of each included paper is vital to confirm inclusion of the studies that met the criteria, and to ascertain integrity and validity of the data in the studies. We used the Critical Appraisal Skills Programme (CASP) to appraise the quality of the included studies (Lachal et al., 2017). This checklist consists of 10 questions which are used to assess quality of qualitative studies for value and trustworthiness.

To ensure the rigour of the assessment was met, LR examined the full texts of the potentially relevant papers individually using the first two questions of the CASP as an initial threshold which led to the screening out of a further five ineligible studies based on their poor quality (unclear qualitative research methods, or justification and /or explanations). Ineligibility was confirmed with PL and KM (Figure 1).

We adopted a three-point rating system for each of the remaining 8 CASP questions (Moolchaem et al., 2018). The three-point scale ranges from one to three: one is a weak score for articles which little or no justification; two is a moderate score for articles with some justification and/or explanation but it is not well – elaborated; and three is a strong score for articles with good justification and strong evidences to support their explanation. To ensure the consistency of the rating of the articles, three studies (Oxlad et al., 2008 ; Elmir et al., 2010; Mackenzie, 2014) were randomly selected and were independently evaluated by the all three reviewers based on the remaining 8 CASP questions Any differences were discussed and resolved leading to a consensus score. The score range of each article was 8-24. To allow easier comparison of quality of reviewed articles, scores 8-15 were considered as weak, 16-23 as moderate and 24 as strong (Table 1). 

## Results

In this study from among 324 found articles, 28 articles were found to be relevant to the aims of this study after quality appraisal. The characteristics of the 28 reviewed articles are be seen Table 2. Most of the studies were conducted in New South Wales, with a small number of studies undertaken in other states in Australia. In the 28 reviewed articles, 904 Australian women’s experiences of breast cancer and associated treatments were reviewed. 

Using thematic analysis, similar experiences before and during the treatment were extracted from the 28 reviewed articles, combined and grouped into five themes and thirteen sub themes (Table 3) (Braun and Clarke, 2013). Examples of excerpts for each of these emergent themes can be found in Supplementary Table 1.

*Theme 1: Breast cancer diagnosis and women’s responses*

In this meta-synthesis, many women expressed feelings of shock, being overwhelmed, emotional upset, and disbelief at the point of their diagnosis. Some were surprised they were diagnosed with cancer despite maintaining a healthy life style such as good diet, having regular mammograms, not smoking or drinking and not experiencing high level of stress (excerpts 1.1 and 1.2). Many women reported they experienced emotional distress on hearing what their treatment would be in the beginning (excerpts 1.3). Despite this initial shock, some women attributed to several factors for being diagnosed with breast cancer such as having existing health conditions (excerpts 1.4 and 1.5).

Interestingly, some Chinese women from Kwok and White’s study culturally associated their breast cancer diagnosis as “white women’s disease”. Thus, they did not expect that it would happen to them.

*Theme 2: Emotional burden*

An emotional burden was felt by women in this meta-synthesis as a result of their breast cancer diagnosis. Two common sub themes were: fear of recurrence and thoughts of death, and the impact on pregnancy were observed across several studies (Figure 3). 

Fear of recurrence and death were evident in multiple studies (Oxlad et al., 2008; Mackenzie, 2014; Keesing et al., 2016; Connell et al., 2006; Thewes et al., 2016; Elmir et al., 2010; Kwok and White, 2014; Coyne et al., 2012; Coyne and Borbasi, 2014; Connell et al., 2006; Fisher and Connor, 2012; Kwok and White, 2013; Gibson et al., 2015; Ives et al., 2012; Halkett et al., 2006; Beatty et al., 2008; Powers et al., 2014). In particular, mothers expressed their fear and concern over their children’s future in the event of their death (excerpts 2.1.1). They feared they may not be able fulfil their roles and responsibilities as a mother if they died (Keesing et al., 2016; Connell et al., 2006; Coyne and Borbasi, 2014; Connell et al., 2006; Fisher and Connor, 2012; Ives et al., 2012; Powers et al., 2014). It was interesting to note that women who practised greater mastery over fear of recurrence has less thoughts of pain and death (Thewes et al., 2016). 

Several studies revealed the women‘s illness increased their family’s practical and economic burden leaving them concerned over their family’s financial status (Mackenzie, 2014; Thewes et al., 2016; Kwok and White, 2014; Coyne et al., 2012; Fisher and Connor, 2012; Kwok and White, 2013; Halkett et al., 2006). Two studies by Coyne and Borbasi (2014) and Fisher and Connor (2012) found that some women had thoughts of reducing their family’s financial burden through their deaths.

Impacts on pregnancy were also apparent with women who became pregnant during their breast cancer diagnosis choosing induced abortion so they could start on their treatment immediately so that they could continue to live for their spouse and existing children (excerpt 2.2.1) (Oxlad et al., 2008, Ives et al., 2012; Kirkman et al., 2014). These studies found induced abortion can be more emotionally challenging for first time mothers diagnosed with breast cancer during their pregnancy versus non-first-time mothers with existing children (excerpt 2.2.2). There was also a natural tendency for first time mothers to worry of being able to conceive again after these abortions. For other first-time mothers who declined abortion, their cancer treatment commenced during their pregnancy. These women were worried about the effects of diagnostic tests and treatments on their unborn child (excerpts 2.2.3). Other first- time mothers were so concerned with the effects of treatment on their unborn child, they chose to start on their treatment immediately after childbirth (excerpt 2.2.4).

*Theme 3: Seeking treatment and challenges*

It was apparent from 12 studies that women wanted to be part of the decision-making process which impact their fertility (Kwok and White, 2014; Kirkman et al., 2012; Lawler et al., 2010; Perz et al., 2013; Smith et al., 2017; Fisher and Connor, 2012; Kwok and Koo, 2017; Ives et al., 2012; Halkett et al., 2006; Halkett et at., 2014; Powers et al., 2014; Kirkman et al., 2014). They often leveraged the internet to “verify information” with information provided by their healthcare providers, to ensure they correctly understood the information before making decisions (excerpts 3.1.1 and 3.1.2). 

However, due to poor English proficiency, some Chinese women felt dependent on others to help them make treatment choices (excerpt 3.1.3). (Kwok and White, 2014; Kwok and Koo, 2017; Kwok and White, 2013). Support groups particularly for Chinese immigrant women were therefore seen as an important source of emotional and culturally tailored information which helped them to decide on breast cancer treatments (excerpt 3.1.4) (Kwok and White, 2014; Lawler et al., 2010; Kwok and White, 2013). 

Making the right treatment decision with an intent to cure sometimes resulted in social distress which such as impact on finances and careers, which resulted in lower quality of life for many women in this meta-synthesis. An important challenge around a breast cancer diagnosis that emerged from this meta-synthesis is the financial implication of treatment. Many women funded the treatment themselves (excerpt 3.2.1). While married women faced financial difficulties for themselves and for their families (excerpt 3.2.2), younger unmarried women face unique financial difficulties including interruptions to career progression (excerpt 3.2.3). 

Several studies highlighted the impact of breast cancer on body image. Many women receiving breast cancer treatment experienced changes in their physical appearance which left them shocked at the emergence of their “new identity” (Oxlad et al., 2008; Mackenzie, 2014; Perz et al., 2013; Smith et al., 2017; Fisher and Connor, 2012; Beatty et al., 2008; Powers et al., 2014; Shaw et al., 2016; Kirkman et al., 2014; Anderson et al., 2011). 

Women who had a better acceptance of their body image coped better with cancer with their “new identity”. Women who had a poorer conceptualisation of their body image had the potential to negatively impact themselves - physically and psychologically, as well as on the well-being of their relationships with others (excerpt 3.3.1 and 3.3.2) (Peterson et al., 2016). In another study by Shaw et al. (2016), half of the women experienced negative body image which affected their self-confidence in entering into a new relationship (excerpts 3.3.3 and 3.3.4). Kwok and White (2014) found some of their Chinese participants did not place much importance on their “new identity” and were less concerned about their body image, instead placing a greater emphasis on their survival so that they could continue their role as a mother (excerpt 3.3.5). 

Younger women are often diagnosed with more aggressive breast cancer and required more intensive therapy than older women (Gnerlich et al., 2008; Sariego, 2010). The study by Anderson et al., (2011), found women <40 years experienced more body image disturbances when compared to older women because of early onset of menopause (excerpt 3.3.6). This led to greater sexual difficulties (excerpt 3.3.7) (Oxlad et al., 2008, Keesing et al., 2016; Perz et al., 2013; Beatty et al., 2008; Anderson et al., 2011). Some women lost their interest in sex as a result of the side effects of their treatments (excerpt 3.3.8) due to lack of libido (Perz et al., 2013; Shaw et al., 2016; Anderson et al., 2011).

Treatment of breast cancer contributed to some women’s fear of being less feminine and unattractive to their partner (Oxlad et al., 2008; Elmir et al., 2010; Smith et al., 2017). Although many young women felt less feminine by the onset of early menopause, two younger women from Anderson et al., (2011)’s study, saw menopause as less distressing compared to the menstrual symptoms (excerpt 3.3.9). This same study also found older women reported greater sleep disruption post treatment compared to younger women (excerpt 3.3.10). Nonetheless, older women accepted menopause positively than the younger women.

Lack of fertility was a challenge around treatment decisions given the often rapid and distressing communication of treatment options following diagnosis. Some women could not focus on the discussion which meant they could not absorb the overwhelming information or they tended to forget the information provided to them. Within this context, women were faced with time constraints in decisions on fertility preservation before they could begin their breast cancer treatment meaning they had to make rapid decisions (Kirkman et al., 2013; Kirkman et al., 2014). However, the women who could not make these rapid decisions displayed “submissive” behaviour by accepting treatment decision exercised by their doctors which as a result affected their fertility (Kwok and White, 2014; Kirkman et al., 2012; Lawler et al., 2010; Smith et al., 2017; Kwok and Koo, 2017). 

Notably, many women were not aware they needed to take steps to preserve their fertility (Kwok and White, 2014; Kirkman et al., 2012; Perz et al., 2013; Kwok and Koo, 2017; Halkett et al., 2006; Halkett el at., 2014; Anderson et al., 2011). Some women reported that fertility preservation was not discussed, with their continuing fertility assumed by doctors (Kirkman et al., 2012; Perz et al., 2013; Fisher and Connor, 2012; Kirkman et al., 2014). Some older women also felt their fertility was assumed by healthcare providers before the start of treatment (excerpts 3.4.1, 3.4.2, 3.4.3 and 3.4.4), leading to inaccurate decision-making resulting in infertility. Further, despite good support and awareness provided by some healthcare providers, other women chose not to participate in any fertility preservation based on their assumption they would regain their fertility after breast cancer treatment (Connell et al., 2006; Perz et al., 2014).

Lack of support for young women in hospital was also reported. Some women shared their frustration at the lack of empathy from male healthcare providers (excerpt 3.4.5), given their lack of personal experience of treatments effect such as hot flushes due to menopause onset. 

Being a mother also meant the protection of children had an impact with many mothers taking steps to protect their children emotionally. Even if mothers were depressed and concerned over the care of their children by the uncertainty of treatment outcomes they also wanted to shield their children from emotional trauma by maintaining a positive attitude in the presence of their children. This protective attitude included false displays of positive strength, shielding children from visits to oncological wards and revealing little information to ensure that the normality of their children’s life was maintained (excerpt 3.5.1). 

Mothers also attempted to protect their children from emotional impact and/or embarrassment due to changes in their body (excerpt 3.5.2). For example, Fisher and Connor, (2012) and Kirkman et al., (2014) found mothers avoided being at their children’s school as their hair loss may impact children’s normality at school. Mothers also carried out their treatments according to their children and family schedules to maintain normality (Mackenzie, 2014, Mackenzie, 2015; Coyne and Borbasi, 2014; Fisher and Connor, 2012; Kwok and White, 2013). 

*Theme 4: Support as coping means*

Women in this meta-synthesis reported that emotional and practical support helped them to cope with the psychosocial impacts of treatments (excerpts 4.1.1 and 4.1.2). 

The majority of women felt a major impact on their ability to carry out their daily household chores, taking care of their children, and attending to their families. For many, their inability to work efficiently due to physical weakness was a constant reminder of their illness. For women with extended families, the parents and sisters of the family usually assisted the women with their household responsibilities. However, for other women such as migrant women, support from extended family was limited and they had to depend on spousal assistance (excerpts 4.1.3 and 4.1.4). .

Many women acknowledged that having a supportive relationship with a spouse (excerpt 4.1.5) was a critical ingredient to their physical and mental health as it buffers a woman’s negative feelings such as feeling of stigma, anxiety and depression (Mackenzie, 2015; Kwok and White, 2014; Coyne et al., 2012; Halkett et al., 2006). Spouses were also reported to hide their feelings (excerpts 4.1.6 and 4.1.7) to provide emotional reassurance to women undergoing treatment (Keesing et al., 2016; Coyne et al., 2012). in other cases, some spouses were initially supportive but became less so in the latter stages of their cancer journey (excerpt 4.1.8), especially with the care of children. 

Interestingly, some women felt better supported emotionally and informationally by their healthcare providers which led to them feeling safer when in hospital (Elmir et al., 2010; Kirkman et al., 2012; Lawler et al., 2010; Halkett et al., 2006; Halkett el at., 2014; Powers et al., 2014). Some women felt that interacting with healthcare providers positively impacted on them which helped them to cope better with their cancer (excerpt 4.2.1 and 4.2.2). On the flip side, some women felt their emotional safety net was removed once their treatment was over (excerpts 4.2.3 and 4.2.5). 

Chinese individuals in this study placed great respect and trust in the expert knowledge of their doctors. Hence, these women place great importance on advice provided by the healthcare providers which in turn helped emotionally support them during their treatment journey (excerpt 4.2.4).

Younger women, on the other hand, found the informational support provided by breast care nurses during treatment was calibrated more for older women and hence, some women sought additional internet support which was more calibrated to their age (Keesing et al., 2016; Lawler et al., 2010; Fisher and Connor, 2012; Gibson et al., 2015; Halkett et al., 2006; Anderson et al., 2011). 

Support networks and groups were another platform to emotionally support women during their diagnosis and treatment. Support groups were also important in gaining some sort of normalcy – reducing stigma and allowing women to freely express themselves without feeling shamed (Luoma and Platt, 2014). However, some women did not want to attend support groups as they were not prepared to share their negative experience of breast cancer, fearing such support groups might make them feel more negative (Oxlad et al., 2008; Shaw et al., 2016).

Although receiving support was seen as being vital in ensuring women cope well during their treatment, it was important for women to take some responsibility for themselves through some self-adjustments. Some women adopted positive self-adjustment strategies (excerpt 4.4.1) whereas others adopted negative self-adjustment strategies journey (Corbin et al., 2013; Powers et al., 2014; Thewes et al., 2016; Shaw et al., 2016; Sinha, 2008; Smith et al., 2017).

*Theme 5: Life after a breast cancer diagnosis*

Studies revealed that participants’ experience of diagnosis and treatment of breast cancer were a turning point in their lives, with the creation of a “new normal world”. This meant recognition and acceptance of breast cancer as now being part of their life, in other words survivorship. 

Women defined survivorship to what life was in its everydayness after diagnosis. This meta-synthesis allowed us to gain insight into this new perspective of their lives and understand their “new normal” world on one’s own terms by creating new meaning of life. This allowed women to have greater appreciation towards life moving forward (excerpts 5.1.1 and 5.1.2). 

Many women developed self-empowerment by taking greater control of their life and reprioritising their life choices. Changing their diet, acquiring greater knowledge through self-education, volunteering to help others, taking part in social events, and choosing not to be in relationship were some of the choices women exercised (excerpt 5.1.3). 

Seeking a new relationship became particularly challenging for single women, with some women trying online dating (excerpt 5.2.1). as they started feeling lonely (Oxlad et al., 2008; Beatty et al., 2008; Shaw et al., 2016). This was necessary as undergoing treatment or recovering post-surgery meant meeting new partners at social events was not be feasible for some women. 

For other women, concerns on future relationships such as fear of rejection from potential partners after the disclosure of a breast cancer diagnosis or physical sequalae such as post-surgery scars/infertility issues as well as trust issues was the motivation for them to use online dating as an alternative strategy so they could feel confident around these issues before meeting potential new partners face-to-face (Perz et al., 2013; Shaw et al., 2016; Kirkman et al., 2014). 

Some women dated selectively by seeking relationships with men with similar cancer experiences, hoping the men would be able to understand their cancer journey more easily. This gave an assurance to women that their potential partners may understand their similar cancer journey and associated challenges (excerpt 5.2.2). 

## Discussion

There is currently no meta-synthesis focused on the lived experiences of symptoms of breast cancer and body image among Australian women diagnosed with breast cancer. This meta-synthesis adds to this evidence-base with an in-depth exploration of how women are emotionally supported during and after treatment. 

Findings from this meta-synthesis suggest most women experience high levels of distress following a breast cancer diagnosis and as a consequence had informational emotional and financial support needs. Being pregnant or a mother added to the complexities around the support needed with unmet needs around fertility decisions highlighted. Many women experienced body image disturbances which impacted on them emotionally and their relationships with others. However, many women reported received support from their spouses or healthcare providers. 

While some of the women included in this meta-synthesis opted to receive treatment during their pregnancy, many chose to start treatment after the birth of their child, fearing possible harm to their unborn baby. These findings show mothers are concerned for their unborn child above all else, including their own health, demonstrated through the value they place on the child (Larson, 1998). Delays in treatment due to the possible risks to unborn children, are at odds with current evidence which suggests chemotherapy only threatens abnormal foetal development and miscarriage if given in the first trimester (Kimby et al., 2004; Amant et al., 2012). However chemotherapy given in the second and/or third trimester may be associated with low birth weight. Choice of treatments in certain stages of pregnancy and concerns on breast cancer should be discussed and be tailored to the individual using a multidisciplinary approach where the team should be focussed both breast cancer treatment and pregnancy care so that the best outcome can be achieved versus potential poorer outcomes due to delayed treatment.

Concern for children also extended beyond pregnancy, with mothers aiming to ensure daily normal routines were continued with minimal disruption to children during treatment. This was to ensure children were provided with caring and protective environments while not being exposed to emotions and illness. In Barnes et al., ’s study, parents gave clear reasons for telling or hiding from their children about their diagnosis. Further, preventing children from becoming distressed and preservation of family time due to uncertainty for the future were reasons for withholding information about illness (Asbury et al., 2014). Additional support for mothers following their diagnosis may be warranted through the use of patient education for parents on managing communication with children after diagnosis and during treatment. In addition, support programmes could be extended to offer children and the wider family to facilitate greater support of ‘normal’ routine in families. 

Many women in this meta-synthesis reported regrets of not pursuing fertility treatment either due to feeling unprepared or lack of appropriate and timely information to make decisions around choice of treatment. The reported low rates of discussion on fertility preservation with healthcare providers highlights the unmet needs of patient around their fertility. Women also indicated that assumptions around their fertility should not be made by healthcare providers even if they are trying to initiate treatment quickly as quality of survivorship and increase distress can be negatively affected due to loss of fertility. Healthcare providers should also have an understanding about the role of women’s concerns and fertility and should use their experiential knowledge coupled with patient-education tools to inform and shape a patient’s right choice of treatment decision.

Expediency of treatment to optimise good outcomes can also affect women’s sexuality and self-esteem due to body image disturbances, which may ultimately affect quality of survivorship particularly for women seeking new relationships. As different women have different pace of adjustments, healthcare providers must give attention to these adjustments. It is likely women’s body image disturbances are related to sexual problems as a result of treatment for breast cancer. It is also possible each woman’s sexual adjustment is different and is dependent on body perceptions together with her sexuality (Sheppard and Ely, 2008). In western cultures, women generally have a strong attachment to their breasts as they define sexuality and femininity (Schmied and Lupton, 2001). Lack of sexual adjustment causes lower self-esteem and altered self-concept of body resulting in negative body image which may affect existing relationships or ability to form relationships. Tailored counselling surrounding body image disturbances unique to each women’s situation could be made part of treatment programmes to assist women in understanding her feelings towards her diagnosis (Nápoles-Springer et al., 2008).

Fortunately, many women described support networks that can help them cope with their diagnosis. For example many of the women in this meta-synthesis acknowledged their spouse was their main caregiver who took care of them and helped them to cope with their illness (Hunt, 2003). Caregivers form an important part of cancer team care and the relationship between caregiver and patient is intertwined (Litzelman et al., 2016). Some women also acknowledged their spouses would try to additionally support them by hiding their own feelings despite being distressed by the breast cancer diagnosis and treatment (Ohayon et al., 2010). Negative psychological distress may negatively impact on the caregiving experience, however, leading to poorer health outcomes for the patient when caregivers feel overwhelmed or burdened. Although studies have highlighted how women receive support from their spouses, there are no findings which demonstrate how spouses are supported in protecting women from psychological distress. This is an important of lack of support for spouses which may have clinical implications on the well-being of women undergoing breast cancer. Findings from this meta-synthesis suggest recognition of the emotional needs of spouses and other family members in conjunction with the patient’s cancer experience is needed. Potentially, family counselling programmes could become part of practice guidelines. 

Positively, many women also felt emotionally supported and reassured by their healthcare providers during treatment. Other women, however, highlighted low rates of discussion for example around and felt rushed in treatment decision-making by healthcare providers following their diagnosis due to limited time frames. There appear to be inconsistencies in the emotional and informational support received at the point of diagnosis and during treatment. Clear guidelines on how healthcare providers support women better emotionally appear needed for example by giving them adequate space to make decisions despite short timeframes and by aiming to understand each patient’s contextual needs.

Nonetheless many women in this meta-synthesis believe the support provided by healthcare providers during breast cancer diagnosis promoted well-being and coping (Arora et al., 2007; Street, 2007). Conversely, however, Landmark et al.,’s study (2002), found there could be increased emotional burden among women with a poorly communicated diagnosis and where information related to diagnosis and treatment was poorly coordinated. This study also found that one third of healthcare providers were not trained to communicate women’s diagnosis appropriately. Communication training programmes for healthcare providers to enable them to deliver diagnoses should therefore be obligatory for those working in oncology settings. 

In conclusion, although many women were emotionally supported following their diagnosis, there are still areas where women could be better supported. This may include support when having to tell children of their breast cancer diagnosis, provision of ongoing support for caregivers of women with breast cancer, provision of consistent support at the time of diagnosis and during treatment as well as standard discussions of fertility in a timely manner by healthcare professionals.

**Table 1:** Quality Appraisal of Reviewed Papers

Author/ Year	Clear aim	Appropriate methodology	Appropriate research design	Appropriate recruitment strategy	Data collection addressed research issue	Consideration of researcher and participant relationship	Ethical issues	Sufficient rigor of data analysis	Clear findings statement	Valuable research	Total score
Oxlad et al., (2008)	Yes	Yes	3	2	3	2	3	2	3	2	20
Mackenzie (2014)	Yes	Yes	3	3	3	3	3	3	3	2	23
Keesing et al., (2016)	Yes	Yes	3	2	3	1	3	2	3	3	20
Connell et al., (2006)	Yes	Yes	3	2	2	2	2	3	3	3	20
Thewes et al., (2015)	Yes	Yes	2	2	2	1	2	2	3	3	17
Elmir et al., (2010)	Yes	Yes	3	2	2	2	3	2	3	2	19
Mackenzie (2015)	Yes	Yes	3	2	3	1	2	2	3	3	19
Kwok et al., (2014)	Yes	Yes	3	2	3	2	3	3	3	3	22
Coyne et al., (2012)	Yes	Yes	1	3	2	1	2	2	3	3	17
Stefanic et al., (2015)	Yes	Yes	3	2	2	1	2	3	2	3	18
Kirkman et al., (2012)	Yes	Yes	1	2	3	2	2	2	3	3	18
Lawler et al., (2010)	Yes	Yes	3	2	2	1	3	2	3	3	19
Coyne et al., (2014)	Yes	Yes	3	2	2	2	3	2	2	2	18
Connell et al., (2006)	Yes	Yes	1	2	2	2	3	3	3	2	18
Perz.et al., (2013)	Yes	Yes	2	1	2	1	2	2	2	2	14
Smith et al., (2017)	Yess	Yes	1	2	2	1	2	2	2	3	15
Fisher et al., (2012)	Yes	Yes	3	2	2	1	3	2	2	1	16
Kwok et al., (2017)	Yes	Yes	3	2	2	1	2	2	3	3	18
Gibson et al., (2015)	Yes	Yes	1	2	2	1	2	2	3	3	16
Ives et al., (2012)	Yes	Yes	3	2	3	2	3	2	3	3	21
Halkett et al., (2006)	Yes	Yes	3	2	2	1	2	2	3	2	17
Beatty et al., (2008)	Yes	Yes	2	3	3	2	2	2	3	3	20
Halkett el al., (2014)	Yes	Yes	3	2	2	1	2	2	2	3	17
Powers et al., (2014)	Yes	Yes	3	2	3	2	2	3	3	3	21
Shaw et.al., (2016)	Yes	Yes	3	2	3	1	2	3	3	3	20

1, offer little to no justification or explanation for particular issue; 2, address the issue but do not fully elaborate; 3, extensively justified and explained; Total scores: weak score, 8 to 15; moderate score, 16 to 23; strong score, 24.

**Figure 2:**
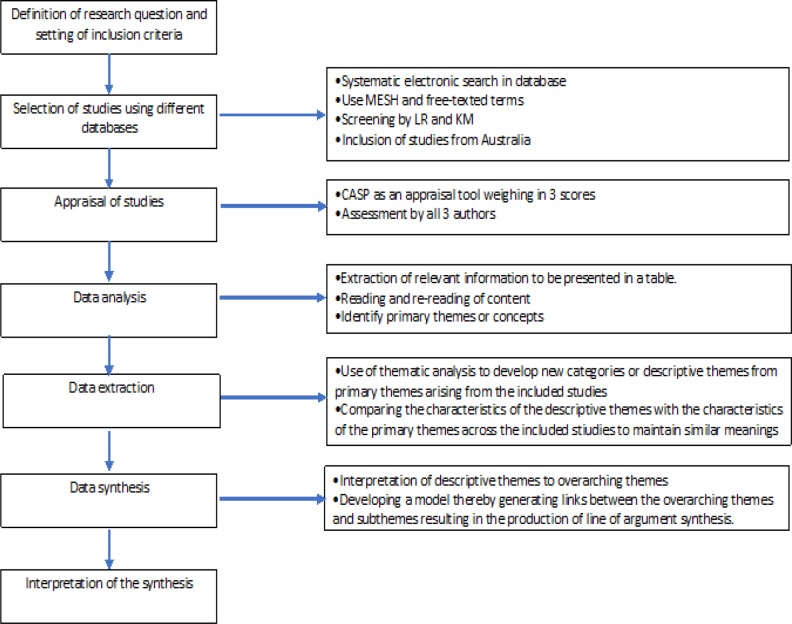
Study Flow Chart

**Table 2:** Emerging Themes and Sub-Themes

Overarching Themes	Sub-themes	Descriptive themes	Primary themes
Challenges associated with breast cancer	Inevitable role as a mother	Protective attitude towards children	Avoid presence at children’s school, shield children from hospital, reveal little info on breast cancer and false display of positive strength to children.
		Continued role as a mother	Treatment plans according to children / family schedule, domestic duties, and communication with children on BC; breast feeding
		Gestational Pregnancy	Abortion, choice of surgery and unborn child’s health, deferred treatment
	“Who am I?”	Body image	Physical change, Psychological change (feel less attractive, embarrassed) Social Dysfunction, reluctance to look naked at oneself, loss of identity
		Femininity	Loss of fertility, Loss of breast (s) and Cessation of period
		Sexuality	Loss of sexual interest, Sexual dysfunction and feeling unattractive to partner
“So, you have survived. What’s next?”		Attitude towards life	Resilience and Optimism, Acceptance, Greater appreciation towards life, Meaning of life, Volunteering to help others, Personal growth
		Self- empowerment	Reprioritise life choices/ lifestyles and Acquire greater knowledge (self- education)
Alternative coping strategies		Positive coping strategies	Social distraction, Physical alteration (tattoo, wig), Personal goal based strategies and Cognitive avoidance
		Negative coping strategies	Drug or alcohol and Use of anger

**Figure 3:**
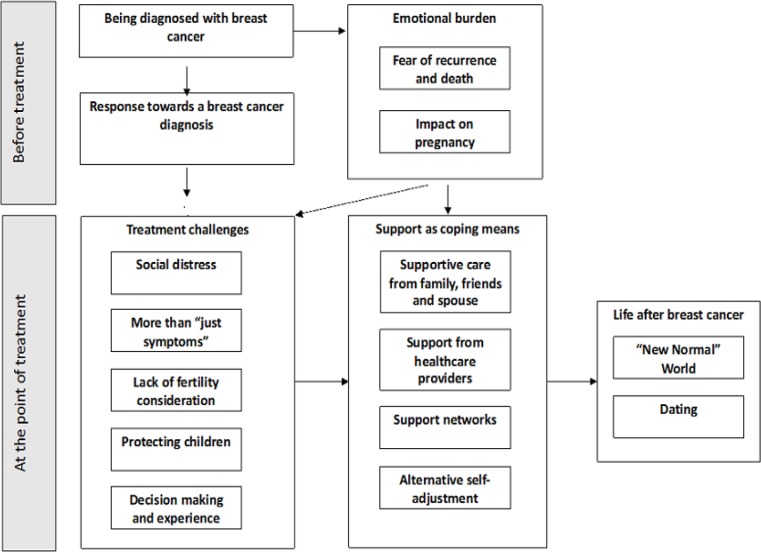
Model of the Lived Experiences of Australian Women Living with Breast Cancer

**Table 3:** Demographic and Methodological Characteristics Included Studies

Author/ Year	Aim/ goal of study	Theoretical orientation/ research approach	Data collection method	Sampling frame	City/ State	Age Range/ Mean	Sample size	Time frame: from diagnosis	Treatment Stage	Type of analysis	Findings
Oxlad et al., (2008)	To identify the current concerns and needs of Australian women who had recently completed primary treatment for breast cancer in order to develop a workbook-journal for this population	Not specified/ Qualitative	Focus group discussion; 10 open-ended interviews	Self-selection	Adelaide, South Australia	36 – 68	10	7 – 51 months	Varied treatment for early stage breast cancer	Interpretative	5 themes: Post-treatment concerns and needs. Australian women’s self-identified current concern and needs following primary treatment, 1. Physical sequelae of treatment, 2. Intimacy issues, 3. Fear of recurrence, 4. Benefit finding 5. Optimism versus pessimism about the future. Completion of primary treatment is clearly a time when women report a number of information/ support needs. Health professionals do not seem to meet their needs. Some women wanted to attend support group and others psychosocial support.
Mackenzie (2014)	To increase understanding of how mothers diagnosed with breast cancer while in the paid workforce experience and manage their multiple demands of taking care of themselves, their children and their paid work	Critical feminist epistemology/Qualitative	In-depth semi-structured interviews	Not specified	Adelaide, South Australia	28-52	32	N/S	Undergoing or completed treatment	Interpretative	Key factors that influenced mothers' decisions: 1. A change in perspective regarding what was important in their lives; 2. Level of support from the workplace and home 3. The extent to which participating in paid work was a financial necessity 4. The extent to which their identity was connected to paid work 5. Ongoing level of pain or fatigue Women in rural location (particularly radiotherapy) found disruptive for children. Most were committed to their employer. Did best to time their treatment to minimise disruption. For some women, family priorities and self-care are breached. The women did more for children rather than asking more assistance from children.
Keesing et al., (2016)	To explore the experiences of women and their partners during early survivorship and contributes a range of insights into the lives of those intimately affected by breast cancer.	Bio- psychosocial framework/ Qualitative	Dyadic in-depth interview	Purposive	Perth, Western Australia	35-70	8 (plus 8 spouses/ partners)	6 months – 5 years	Completed treatment for breast cancer (excluding adjuvant hormone treatment)	Thematic	3 themes: 1. a disconnection within the relationship by women 2. reformulating the relationship 3. support is needed to negotiate the future of the relationship Women felt their physical, psychological and emotional needs were largely undervalued by their usual medical supports Partners of women reported many unmet needs and were unaware of where they could obtain assistance to help them. Acknowledge that breast cancer experience must be shared and accepted that there are changes to intimate relationships.
Author/ Year	Aim/ goal of study	Theoretical orientation/ research approach	Data collection method	Sampling frame	City/ State	Age Range/ Mean	Sample size	Time frame: from diagnosis	Treatment Stage	Type of analysis	Findings
Connell et al., (2006)	To better understand their concerns and needs.	Constructio-nist epistemology/Phenomenology	Semi-structured interview	Convenience	Queensland	29–40 / 37	13	5–37 months (mean = 26 months)	Not specified	Interpretative	Issues and concerns: 1. Perceptions of fertility changed over time. 2. Contraception issues 3. recurrence fears related to pregnancy and breastfeeding after breast cancer. 4. Decisions related to unplanned pregnancies and breastfeeding Health professionals need to communicate such findings clearly to help quash unnecessary concerns of young women with breast cancer. Mixed feelings about wanting a child
Thewes et al., (2015)	To qualitatively explore the strategies used by younger breast cancer survivors to cope with FCR and whether women with low, medium and high levels of FCR employ different coping strategies.	Transcen-dental realism/ Qualitative	Telephone interviews	Not specfied	Australia	32 - 45	28- Australian 10- Canadian	Diagnosed with stages 0–II breast cancer at least 1 year prior 2 and 11 years post-diagnosis ≤45 years at the time of diagnosis	Not specified	Deductive and Inductive	3 Themes: 1. Coping 2. Differences in coping between women with low versus moderate to high FCR 3. Worst fears Women with high FCR might engage in time-consuming health behaviours. Women with lower FCR describe a greater repertoire of strategies and a greater sense of mastery over FCR.. By contrast, women with moderate to high levels of FCR reported using fewer coping strategies and described their coping efforts in a manner. Fear of death is common amongst many young women with breast cancer.
Elmir et al., (2009)	To generate insight into younger women’s experiences of recovery from breast cancer-related breast surgery and to contribute to the knowledge base for clinicians practising in this field	Not specified/ Narrative phenomenology	Semi-structured- interview	Not specified	New South Wales	31-48	4	Not specified	Varied treatments (all had chemo)	Thematic	4 themes: 1. It absolutely encompassed me,’ 2. ‘Being overwhelmed,’ 3. ‘Living with fear and uncertainty’ 4. ‘Finding strength within.’
Mackenzie (2015)	To examine women’s experiences of enablers and constraints to physical activity participation after being diagnosed with breast cancer while mothers of dependent children	Not specified/ Qualitative	Semi-structured in-depth interviews	Not specified	Adelaide, South Australia	28-52	36	21- diagnosed within 5 years on the interview	Not specified	Interpretative	Importance of physical activity and partner support QoL and depression scores were consistent with community rates although anxiety scores were higher. Approximately two thirds of survivors reported at least one unmet need, most frequently concerning existential survivorship issues, thereby highlighting the unique needs of survivors.
Author/ Year	Aim/ goal of study	Theoretical orientation/ research approach	Data collection method	Sampling frame	City/ State	Age Range/ Mean	Sample size	Time frame: from diagnosis	Treatment Stage	Type of analysis	Findings
Kwok and White (2014)	Explores Chinese- Australian women’s perceptions of the meaning and experience of a breast cancer diagnosis, treatment and coping mechanism	Not specified/ Qualitative	Focus group interviews	Not specified	New South Wales	40-69	23	Not specified	Undergo varied treatments (all had surgery)	Structured content	How these women experienced their illness and attempted to capture the wholeness of their experiences, particularly the psychological and physical impact of breast cancer. These women found the experience isolating and distressing, factors that were compounded by the lack of culturally sensitive resources and information.
Coyne et al., (2012)	To examine the role and strengths of the family when supporting the younger woman (<50 years) after a diagnosis of breast cancer. The perspectives of women and family members were sought.	Resiliency Model of Family Stress/ Qualitative	Semi structured interview and phone interview	Not specified	Queensland	35-46	14 (plus 11 family support persons)	Not specified	Varied treatments	Thematic	roles of family changes: ‘just being there’, ‘paradox of help’ and ‘buffer from society’. A secondary theme related to support, specifically ‘the changing role of support for family members’, highlighting the strengths and experiences of family.
Stefanic et.al., (2015)	To better understand the nature of situational goal-based coping in response to personal goal interference encountered across the six months following surgery for early-stage breast cancer.	Dual-process model/ Qualitative	Semi-structured telephone interview and survey	Not specified	Victoria	39-76 (mean =62)	36	Not specified	Varied treatments	Thematic	Early-stage breast cancer patients utilised goal-based coping in response to many instances of goal-specific interference encountered during the study period.
Kirkman et al., (2012)	Young women’s experiences of cancer care and the ways in which their health care providers managed their concerns about fertility and childbearing.	Not specified/ Qualitative	In-depth interviews by face-to-face and telephone interviews	Convenience	New South Wales	26–45	10	Diagnosed at 25–41 years	At least 1 year post-diagnosis	Thematic	Thematic analysis revealed that all women, including one who chose to be child-free, valued fertility and motherhood. All wanted health care providers to communicate fertility options and avoid assumptions about women’s fertility desires while working to extend each woman’s life.
Lawler et al., (2010)	To explore and examine experiences and perceptions of follow-up care (medical and psychosocial) after active treatment for breast cancer among women living outside major Australian cities	Not specified/ Qualitative	Semi structured telephone interviews	Purposive	Queensland	35 – 69 (mean = 49)	25	Diagnosed (10 months - 5 years Mean = 2.5 years	Varied treatments	Constant comparative	Explore and examine experiences and perceptions of follow-up care (medical and psychosocial) after active treatment for breast cancer
Coyne and Borbasi (2014)	To explore the experience of a diagnosis of breast cancer for women under the age of fifty	Feminist epistomology	in-depth interviews	Purposive	Queensland	29 - 43	6	Diagnosed with BC last 12 months;	Varied treatments	Thematic	Focus on young women and the impact it has on their social situation and their family.
Author/ Year	Aim/ goal of study	Theoretical orientation/ research approach	Data collection method	Sampling frame	City/ State	Age Range/ Mean	Sample size	Time frame: from diagnosis	Treatment Stage	Type of analysis	Findings
Connell et al,.(2006)	Exploring the experiences of young women with breast cancer, extending available knowledge in this area to provide insights into the relative importance assigned to various issues and concerns	Not specified	In-depth semi-structured face-to-face interview or telephone interview	Convenience	Queensland	23-43 (mean = 36)	35	4-39 months (mean=21 months)	Varied treatments	Content (Miles and Huberman)	Main results: Second to fear of recurrence and future uncertainty, children and family were the most commonly reported major personal concern. Consumer-related issues and concerns for children and family were equally reported as the greatest general concern of young women with breast cancer. The greatest unmet need of participants was support.
Perz et al., (2013)	To examine the subjective experience of changes to fertility status in a large sample of women with breast cancer living in Australia, using quasi-qualitative methods	Not specified/ Qualitative	Online survey by 2 open-ended questionnaires	Not specified	New South Wales	Mean 54.1	381	Diagnosed mean = 3.9 years	Completed treatment and continue varied treatments	Thematic	5 themes 1. ‘Negative responses to infertility and early menopause’; 2. ‘Sexual changes associated with menopause and infertility’; 3. ‘Uncertainty and anxiety about fertility status’; 4 ‘Information and fertility preservation’ 5. ‘Acceptance of the end of fertility’
Smith et al., (2017)	The purpose of this study was to develop an overview of perceptions and experiences of women undergoing taxane-based treatment for early breast cancer.	Not specified/ Qualitative	5 Focus group discussions with semi-structured interview	Not specified	Victoria	35-64 (median = 50)	25	1-89 months (mean = 25 months)	Completed taxane- based chemotherapy treatment between March 2008 and November 2015.	Thematic	Explore participants’ perceptions and experience of madarosis during and following chemotherapy and identified issues associated with impact of madarosis on quality of life (QoL); 7 themes 1. timing of regrowth and permanent changes, 2. meaning/importance of eyebrow/eyelashes 3. preparedness/information given, 4. impact of the hair loss of self, 5. impact of hair loss on others, 6. physiological side effects of loss of eyebrows/eyelashes, 7. management of loss of eyebrows/eyelashes
Fisher and Connor (2012)	To explore the impact of breast cancer on the identities of young women as ‘‘mothers.’’	Social constructivist/ Qualitative	In-depth interview	Purposive	Western Australia	31-42 (mean = 35)	8	5 months – 7 years	Varied treatments	Interpretative	4 themes not mutually exclusive 1. Diagnosis and disruption 2. Maintaining normality 3. Continuing the mothering role 4.Experiencing survivorship Mothering role (responsibilities towards children and stability of family) be acknowledged by health professionals.
Author/ Year	Aim/ goal of study	Theoretical orientation/ research approach	Data collection method	Sampling frame	City/ State	Age Range/ Mean	Sample size	Time frame: from diagnosis	Treatment Stage	Type of analysis	Findings
Kwok and Koo (2017)	To fill in a gap in the literature by examining the extent to which cultural values and language affect participation in TDM by Chinese women in Australia	Confucian philosophy/ Qualitative	3 Focus groups discussion	Not specified	New South Wales	35-68 (mean = 56)	23	diagnosed with cancer in the last 6 months;	all undergone surgery	Content analysis	How these women made their treatment decisions - 4 types of decision makers 1. the patient as an active decision maker, 2. the patient as a passive decision maker, 3. the patient as a reluctant decision maker 4. the patient as a reluctant passive decision maker. Language barriers, cultural expectation of doctor’s role and family role in Chinese culture appear as influential factors in TDM process among this group of women.
Kwok and White (2013)	To explore the perceptions of information needs and social support among Chinese-Australian breast cancer survivors and how these resources impacted their cancer experience.	Not specified/ Qualitative	3 semi-structured focus groups interviews	Not specified	New South Wales	35-68 (mean =56)	23	diagnosed with cancer in the last 6 months;	All undergone varied treatments	Content	4 Themes for information needs were identified as (1) using linguistically appropriate information, (2) the need for culturally sensitive information for the management of expect- ed side effect and promotion of recovery and (3) the need for information on signs and symptoms of recurrence
Gibson et al(2015)	Examine women’s talk of having breast cancer through the lenses of ‘healthism’ and ‘risk management’ and consider the possible productive and restrictive effects of these neo-liberal discourses for women in making sense of their illness	Feminist and post-structuralist theory/not specified	Semi structured telephone interview	Not specified	Adelaide, South Australia	29 – 72 (mean = 55)	27 Lesbian-4 CALD -10 Rural -15	Not specified	Not specified	Multimodal critical discursive	Thematic discourse: 1. Health and risk talk about breast cancer 2. Accounting for the cancer diagnosis 3. Always healthy 4. Being ‘at risk’ 5. Practising control and responsibility
Ives et al.(2012)	To explore the psychosocial experiences of pregnancy in women diagnosed with breast cancer during or shortly after pregnancy.	Not specified/ Retrospective qualitative	Semi structured interview	Not specified	Western Australia	< 45	15	Diagnosed with GBC after 1 January 1982;	Not specified	Content	difficulty adjusting to BC and pregnancy; high level of stress and anxiety
Author/ Year	Aim/ goal of study	Theoretical orientation/ research approach	Data collection method	Sampling frame	City/ State	Age Range/ Mean	Sample size	Time frame: from diagnosis	Treatment Stage	Type of analysis	Findings
Halkett et al.(2006)	Provides an understanding of the broad range of decisions with which women may be faced, and presents a new interpretation of what the experience of making decisions is like for women diagnosed with breast cancer.	Heidegger, Gadamer and van Manen/ Hermeneutic phenomenology	In-depth interview face-to-face	Purposive	Western Australia	39–77/ mean= 59	18	EBC women	All undergone surgery and completed adjuvant treatment	Interpretative	Experience of making decisions 5 themes: 1. being challenged, 2. getting ready 3. surviving 4. sharing the challenge 5. interrogating the future.
Beatty et al.(2008)	To qualitatively identify the concerns and needs of Australian women recently diagnosed with breast cancer	Not specified/ Qualitative	4 patient-based focus group discussions	Not specified	Adelaide	Mean=53.5	19	EBC women diagnosed last 12 months	Varied treatments and completed treatment	Thematic	5 areas of concerns: (i) coping with side-effects; (ii) dealing with self-concept change; (iii) stress and adjustment reactions; (iv) having to manage others’ unhelpful beliefs, expectations and emotions; and (v) issues with survival and growth
Halkett el at. (2014)	To explore patients’ perspectives of the role of the breast care nurse	Not specified/ Hermeneutic phenomenology	In-depth interview	Not specified	Adelaide	39-77 (Mean = 59)	18	Not specified	EBC women who received treatment in 2003 and completed	Thematic	BC nurse provides important support during EBC;
Powers.et al. (2014)	To develop a better understanding of women’s subjective experiences of breast cancer post-treatment, and the social contexts of these experiences in order to add to public discourse about this topic, and to provide valuable information to both those who treat the disease and those who experience it firsthand.	(N.P.) experience of breast cancer/ Phenomenology	Semi-structured in-depth interview	Convenience	New South Wales	49-73	9	Diagnosis last mean =8 years ago	Women who completed all BC treatment	Thematic	survivorship can remain challenging and can present adverse psychosocial implications for women and those close to them
Shaw et al. (2016)	To explore women’s dating experiences after breast cancer, including any challenges that they experienced to forming a relationship and their ability to cope with dating-related anxieties.	Not specified /Grounded theory	semi-structured interviews	Not specified	New South Wales	27-74 (Mean = 47)	22	12-81 months / mean = 38.6 months	In a heterosexual relationship	Constant comparison	7 themes emerged: 1. women’s decision to consider dating 2 ability/ desire to commence a new relationship 3. cancer-related disclosure 4. changes to intimacy and sexuality 5. body image difficulties 6. changing values 7 trusting a new partner.

## Limitations

This meta-synthesis has several limitations. First, only English language studies were included, It is possible cultural studies in other languages were overlooked. Second, most of the included studies did not investigate emotional support needs for caregivers. This meta-synthesis could therefore not consider the whole family as a unit of care, just the patient. Third, most women included here were mothers, with many reporting higher distress levels and concerns about their children therefore findings may not be generalisable to non-mothers.
